# Recovering More than Tree Cover: Herbivores and Herbivory in a Restored Tropical Dry Forest

**DOI:** 10.1371/journal.pone.0128583

**Published:** 2015-06-01

**Authors:** Iris Juan-Baeza, Cristina Martínez-Garza, Ek del-Val

**Affiliations:** 1 Facultad de Ciencias Biológicas, Universidad Autónoma del Estado de Morelos, Av. Universidad No. 1001, Col Chamilpa, Cuernavaca, Morelos, C.P. 62209, México; 2 Centro de Investigación en Biodiversidad y Conservación, Universidad Autónoma del Estado de Morelos, Av. Universidad No. 1001, Col Chamilpa, Cuernavaca, Morelos, C.P. 62209, México; 3 Instituto de Investigaciones en Ecosistemas y Sustentabilidad, Universidad Nacional Autónoma de México, Antigua Carretera a Pátzcuaro No. 8701, Col. Ex-Hacienda de San José de La Huerta, C.P. 58190, Morelia, Michoacán, México; University of Guelph, CANADA

## Abstract

Intense and chronic disturbance may arrest natural succession, reduce environmental quality and lead to ecological interaction losses. Where natural succession does not occur, ecological restoration aims to accelerate this process. While plant establishment and diversity is promoted by restoration, few studies have evaluated the effect of restoration activities on ecological processes and animal diversity. This study assessed herbivory and lepidopteran diversity associated with two pioneer tree species growing in 4-year-old experimental restoration plots in a tropical dry forest at Sierra de Huautla, in Morelos, Mexico. The study was carried out during the rainy season of 2010 (July-October) in eleven 50 x 50 m plots in three different habitats: cattle-excluded, cattle-excluded with restoration plantings, and cattle grazing plots. At the beginning of the rainy season, 10 juveniles of *Heliocarpus pallidus* (Malvaceae) and *Ipomoea pauciflora* (Convolvulaceae) were selected in each plot (N = 110 trees). Herbivory was measured in 10 leaves per plant at the end of the rainy season. To evaluate richness and abundance of lepidopteran larvae, all plants were surveyed monthly. Herbivory was similar among habitats and *I*. *pauciflora* showed a higher percentage of herbivory. A total of 868 lepidopteran larvae from 65 morphospecies were recorded. The family with the highest number of morphospecies (9 sp.) was Geometridae, while the most abundant family was Saturnidae, with 427 individuals. Lepidopteran richness and abundance were significantly higher in *H*. *pallidus* than in *I*. *pauciflora*. Lepidopteran richness was significantly higher in the cattle-excluded plots, while abundance was significantly higher in the non-excluded plots. After four years of cattle exclusion and the establishment of plantings, lepidopteran richness increased 20 –fold in the excluded plots compared to the disturbed areas, whereas herbivory levels were equally high in both restored and disturbed sites. Restoration with plantings and exclusion of cattle and plantings was shown to be a successful strategy for attracting lepidopterans and cattle exclusion was the main factor explaining lepidopteran diversity.

## Introduction

Intense and chronic disturbance may arrest natural succession, reduce environmental quality and lead to the loss of ecological interactions [1,2]. Ecological restoration aims to accelerate the process of natural succession, in cases where it does not occur [3,4]. Manipulation of natural succession by planting late-successional tree species may increase the richness and density of plants and animals in early-successional environments. However, there are few studies that have evaluated the significance of these techniques for faunal diversity and ecological processes [5–10].

The selective pressure imposed by herbivory determines the survival and fitness of plants [11,12]. Many restoration efforts are hampered by herbivores that may impede plant development; vertebrates in particular have been involved in many restoration failures [13–15]. In tropical systems lepidopterans are very diverse and function as important herbivores since they consume significant quantities of leaf tissue [16,17]. As adults, moths and butterflies participate in the vital process of pollination [18,19]; in fact in tropical dry forests they account for the pollination of at least 10% of the plant species [20]. It is therefore desirable that functional restored ecosystems have a complement of herbivores that will become effective pollinators when they reach adulthood.

Habitat perturbation has shown a significant effect on plant-herbivore interactions [21]; disturbed habitats present greater levels of herbivory when generalist herbivores are promoted [22,23] or, conversely, present reduced herbivory where the main herbivores are specialists and have disappeared following habitat perturbation [24]. Few studies have evaluated the presence of lepidopterans in restoration projects (but see [5,25]) and therefore there is an urgent need to determine whether lepidopterans are able to reach to restored sites and, if so, whether they are able to provide the same ecological services as is the case in natural habitats.

The effect of cattle in dry forest has been evaluated for plants and animals. Cattle browse understory herbs and grasses and also the lower foliage of trees negatively affecting tree diversity [26,27] and bird populations [28,29]. Cattle ranching activities are also known to affect the composition of trees [30], cacti [31], herbs and grasses [32]. Decreases in biodiversity also have a negative effects on ecosystem processes but changes in composition are expected to have higher negative impacts [33]. For example, changes in tree composition in dry forest as a result of cattle ranching can alter seed dispersal patterns [34,35] and also favor the establishment of weeds dispersed by cattle [32,36]. Furthermore, cattle may decrease the size of large cacti, due to trampling, affecting the frugivorous animals that depend on them [31]. Cattle affect dry forest functioning by changing the composition of plants and indirectly through their effects on plant-animal interactions, such as dispersal.

We evaluated Lepidopteran richness, abundance and composition during the rainy season of 2010 (July-November) in a 30 year-old secondary tropical dry forest at Sierra de Huautla in Central Mexico in order to estimate the potential recovery of ecosystem function after 4 years of exclusion of chronic disturbance by cattle. We addressed the following specific questions: 1) How does the lepidopteran larval diversity associated with two pioneer species differ between habitats? and 2) how does accumulated leaf herbivory in two pioneer species differ between three habitats: two restoration settings (plantings and cattle exclusions) and disturbed areas? We proposed the following hypotheses: 1) increased lepidopteran richness and density will occur in the individuals growing in the restoration plantings and the lepidopteran communities will differ in terms of species and habitats and 2) there will be higher accumulated herbivory in the individuals growing in the restoration plantings,

## Materials and Methods

### Study site

This study was carried out close to the town of El Limón de Cuauchichinola (1220 m a.s.l.), located within the Sierra de Huautla Biosphere Reserve (SHBR), in the south of the state of Morelos, central Mexico. The SHBR (18° 20’ 10”, 18° 34’ 20” N and 98° 51‘ 20”, 98° 08‘ 15” W) comprises 59,030 ha, in which the main vegetation type is tropical dry forest. Mean annual temperature is 24.5°C and average total rainfall (average for 1971–2000) is 817.5 mm (CONAGUA, Gerencia Regional Balsas, http://smn.cna.gob.mx/climatologia/normales/estacion/mor/NORMAL17057.TXT), with ~90% of this rainfall occurring between late May and October. Most of the trees shed their leaves during the dry season (November to May). The soils are shallow (< 30 cm in depth) and sandy-loam in texture.

In the SHBR, 939 native species of vascular plants from 478 genera and 130 families have been recorded [37]. Of these, 157 species are trees or shrubs [38]. This forest has one stratum of trees 8 to 12 m in height with convex or flat canopies. Most of the leaves of these trees are compound with small leaflets [39]. In terms of number of species, the most important families are Fabaceae, Poaceae, Asteraceae and Burseraceae. The most common canopy trees are *Conzattia multiflora* (B.L. Rob.) Standl., *Lysiloma acapulcense* (Kunth) Benth., *Lysiloma divaricatum* (Jacq.) J.F. Macbr. (Fabaceae), *Bursera* spp. (Burseraceae) and *Ceiba* spp. (Bombacaceae) [37].

### Land history and description

The landscape of the SHBR comprises a mosaic of primary and secondary dry tropical forest surrounded by agricultural land and small towns. Forty one percent of the area of the reserve has been classified as intact or under good conservation status, whith 22.4% classified as well-conserved, based on aerial images and vegetation surveys conduceted at selected points. The remaining 36.2% presents different degrees of degradation and most of this fraction is used for economic activities [37,40]. In El Limón de Cuauchichinola (referred to hereafter as El Limón), large parts of the forest were cleared > 30 years ago, used for maize cultivation for *ca*. 6 years and subsequently abandoned. Since abandonment, the resulting secondary forest has been used for wood extraction and extensive cattle ranching (G. Pacheco, *pers*. *comm*). In El Limon, 56% of the area is currently covered with intact forest or is under good conservation status, 19% is perturbed dry forest, 12% is secondary vegetation and the remaining 13% is dedicated to agriculture and housing [41]. In this forest, approximately 50% of the organic matter is concentrated within the upper 10 cm of the soil profile (E. Solís, *unpub*. *data*) and the tree density of individuals ≥ 5 cm of diameter at breast height (1.3 m) is 264 individuals/ha from 14 tree species. Of these, *Acacia cochliacantha* (Humb&Bonpl) (Fabaceae), *Ipomoea pauciflora* (Mart&Gal) (Convolvulaceae), *Acacia farnesiana* (Willd) (Fabaceae) and *Mimosa benthamii* (Macbride) (Fabaceae) are dominant [42]. During the rainy season, *ca*. 600 head of livestock forage in this secondary forest (~ 7 head/ha); most of these animals are brought to the site from neighboring towns. Cattle are maintained in farms during the dry season but goats, pigs and horses are left to forage in the forest daily throughout the year. Grazing reduces the biomass in the understory by more than 70% [32]. As part of an experimental restoration project to promote the coexistence of cattle grazing activities and forest biodiversity, eight 50 x 50 m plots were excluded from disturbance in the form of wood extraction and grazing by large domestic livestock since January 2006. Exclusion was accomplished with a fence of 4 lines of barbed wire, with an additional 75 cm of chicken wire netting attached to the lower part of the fence to exclude smaller domestic animals. In 2010 an additional electric fence was installed outside the original fence. Distances among plots ranged from 0.08 to 1.59 km (0.72 ± 0.46; mean ± standard deviation). The closest old-growth forests were located at a range from 0.11 to 0.26 km (0.21 km ± 0.05) from the plots.

Natural succession was manipulated in half of the sites with enrichment plantings of 18 native tree species found in late-successional environments and two early-successional species (for experimental details see [23] [22] [21] [20]). The entire experiment consisted of four plots fenced against cattle where natural succession took place, four plots fenced against cattle with additional plantings and three control plots where cattle freely grazed with some remaining adult trees.

In June of 2010, two early successional species that occur naturally in all of the sites (*Ipomoea pauciflora* [Convolvulaceae] and *Heliocarpus pallidus* [Tiliaceae]) were selected for this study. Ten naturally recruited juveniles of each species (average height 0.5–2 m) per plot were selected in the eight plots. Due to cattle ranching activities, no natural recruitment occurred outside the plots and consequently only adult individuals of the species selected were found.

### Herbivore sampling

Lepidopteran larvae were sampled monthly during the wet season (July—November 2010), when both leaves and larvae are present. We surveyed all the leaves in the chosen trees from. *H*. *pallidus* and *I*. *pauciflora* up to a height of 2 m in order to find lepidopteran larvae. In taller trees, we sub-sampled 3 branches per individual [43]. In each survey, unknown lepidopteran larvae were reared in the laboratory in order to taxonomically identify the adults. When there were several larvae of the same species eating in the same plant only three larvae were taken to the lab. When the larvae were already identified, they were left in the field.

### Herbivory rates

At the end of the growing season, before the trees started to shed their leaves, 10 leaves per individual tree were collected and transported to the laboratory in order to evaluate herbivory rates at the different sites. Each leaf was measured with a portable leaf area scanner (CI-202 Leaf Area Meter–CID, Inc) to calculate the area of leaf removed and accumulated herbivory percentage values were calculated per plant. All sampling procedures and experimental manipulations were approved as part of obtaining the field permit from the General Direction of Mexican Wildlife (permit number SGPA/DGVS/ 02693/10 to Cristina Martínez-Garza).

### Statistical analysis

Statistical analyses for herbivory were performed with a factorial analysis of variance (ANOVA). The dependent variable was the average leaf area consumed per plant and the independent variables were species with two levels (*I*. *pauciflora* and *H*. *pallidus*), habitat with three levels: non-excluded (N = 3 sites), excluded (N = 4 sites) and excluded with plantings (N = 4 sites) and we had ten individuals per species per habitat. The average leaf area per plant consumed was transformed with the arcsine square root of the proportion of herbivory + 0.1 in order to comply with the assumptions for ANOVA.

In order to analyze Lepidopteran diversity, two repeated measures ANOVAs were performed. The dependent variables were richness and abundance of lepidopteran larvae and the independent variables were species identity with two levels and habitat with three levels (exclusions with planting, exclusions with no planting and non-excluded plots). The repeated measure was sampling month, with four levels (July, August, September and October)

To analyze changes in lepidopteran species composition associated with restoration treatment, we also performed a clustering analysis using a distance matrix obtained with Bray-Curtis index of total lepidopteran abundance per species per treatment and plotted the resulting dendrogram showing Bray-Curtis distances and, in order to obtain the tree significance, we performed a Mantel test with 100 permutations using the “vegan” library in R 2.14.0 [44].

## Results

We recorded 868 caterpillars from 64 morphospecies during the four months of study. To date, 15 of these have been identified to family level, four to genus level and nine to species level (S1 Table). We recorded species belonging to 11 families; the highest number of species (nine species) was from the Geometridae, whereas the highest caterpillar abundance (429) was from the Saturnidae. Most of these individuals (422) were from one species: *Arsenura armida*. Only two species were found to be associated with both plants: *Orgyia* sp. (Lymantridae) and *Hypercompe suffusa (*Arctidae).

### Lepidopteran richness

**Fig 1:**
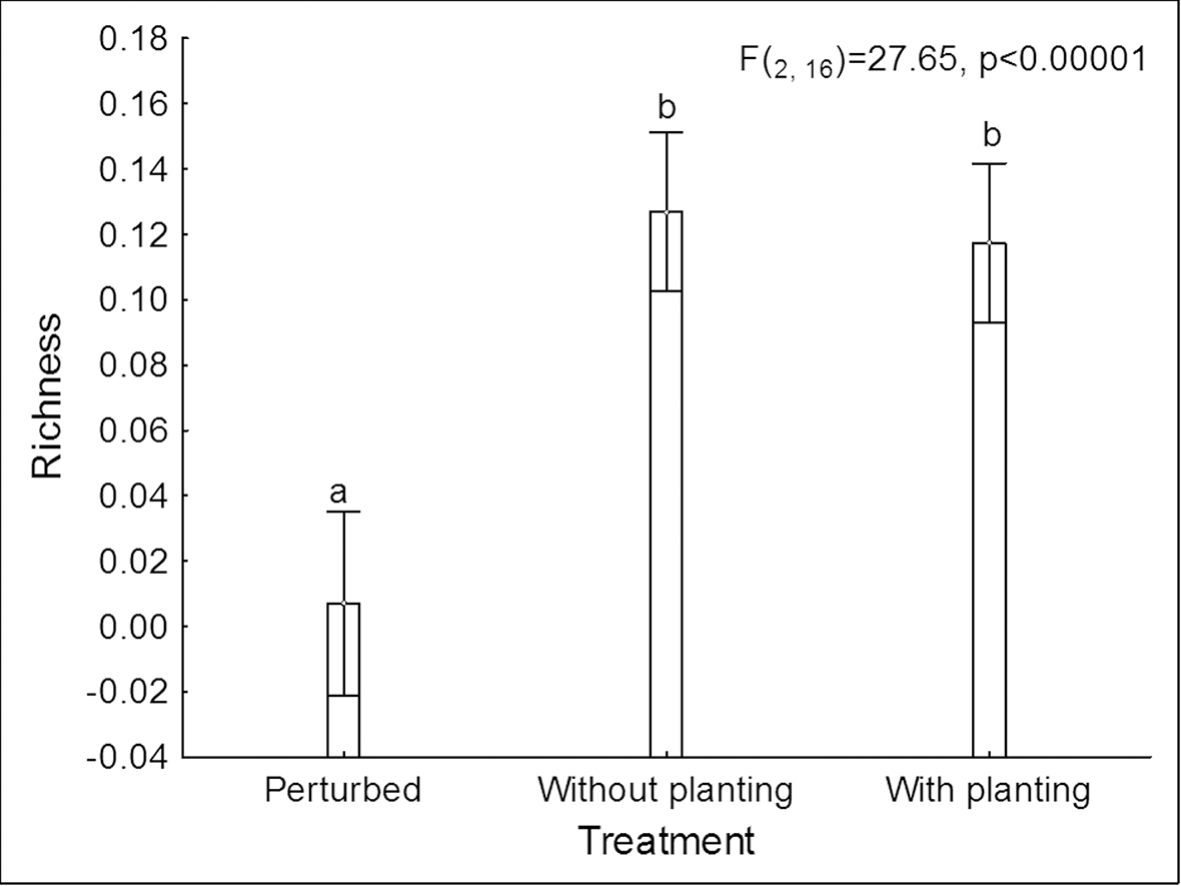
Lepidopteran richness associated with tree species in different experimental treatments. Lepidopteran richness (log10 +1) associated with trees of *I*. *pauciflora* and *H*. *pallidus* in excluded sites, with and without plantations, and disturbed sites with cattle grazing in the tropical dry forest of Huautla, Morelos, Mexico. Vertical lines represent a 95% confidence interval. Different letters represent significant differences evaluated with a post hoc Tukey test.

Richness of lepidopterans associated with *H*. *pallidus* (3.05 ± 0.45 morphospecies) did not differ significantly from those associated with *I*. *pauciflora* (2.09 ± 0.32 morphospecies; F_(1, 16)_ = 3.81, P > 0.07) and this was true for all of the habitats (species* habitat: F_(2,16)_ = 1.21, P > 0.32). However, plants growing in the excluded plots and those growing in the plantings presented 20 times higher richness (3.63 ± 0.45 and 3.31 ± 0.45 morphospecies respectively) than those found in plants growing in the non-excluded plots (0.17 ± 0.08 morphospecies). The *post hoc* Tukey test revealed that excluded plots and plantings showed similar lepidopteran richness and this was found to be significantly higher than that of the non-excluded plots (F_(2,16)_ = 27.65, P<< 0.001, Fig 1).

**Fig 2:**
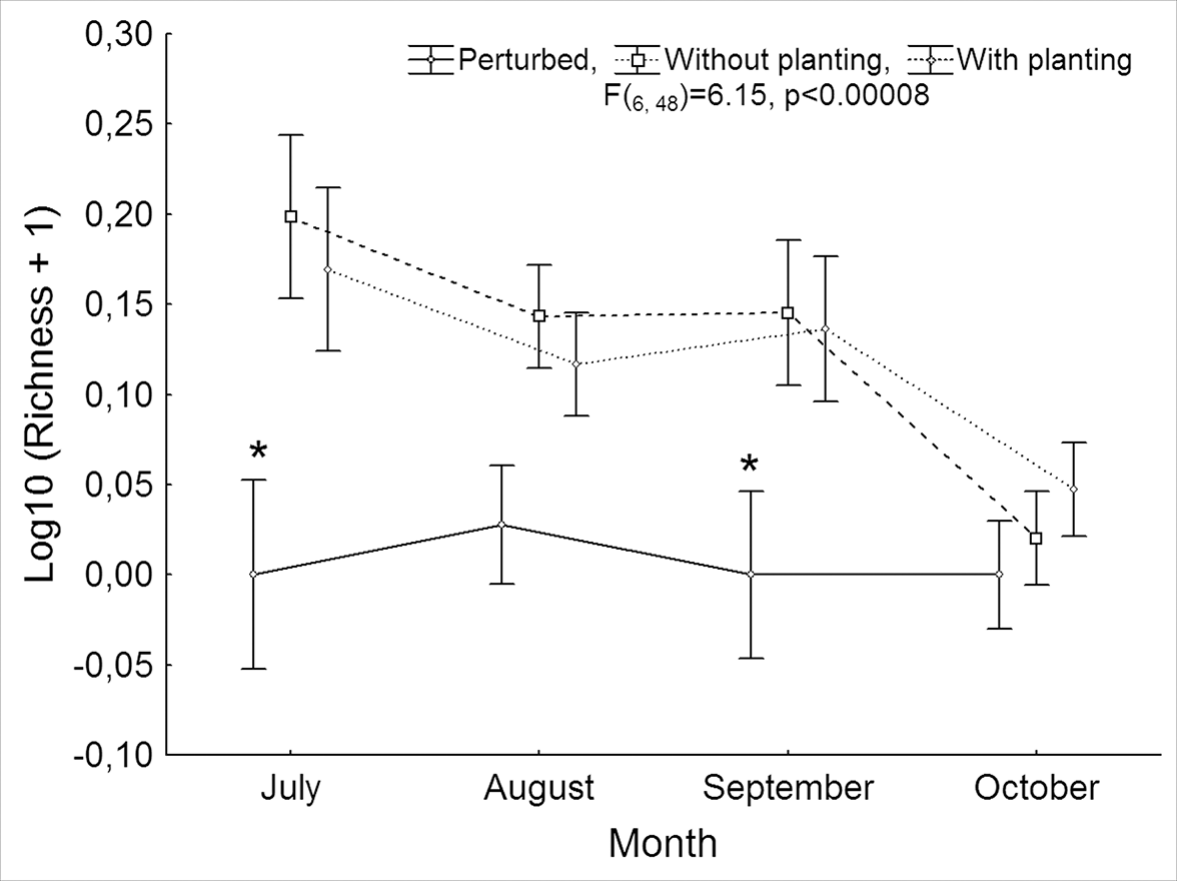
Temporal richness of Lepidopteran larvae. Temporal pattern of lepidopteran larvae richness associated with trees of *I*. *pauciflora* and *H*. *pallidus* in excluded sites, with and without plantations, and perturbed sites with access for livestock during the rainy season from July to October 2010 in the Sierra seasonal forest Huautla, Morelos, Mexico. Vertical lines represent a 95% confidence interval. Asterisks represent significant differences between treatments in the same month evaluated with a *post hoc* Tukey test.

Lepidopteran richness decreased throughout the rainy season (F_(3, 48)_ = 23.91, p<<0.0001). The *post Hoc* Tukey test revealed that lepidopteran richness was similar in July (4.00 ± 0.71 morphospecies), August (2.77 ± 0.37) and September (2.86 ± 0.49) but significantly lower in October (0.63 ± 0.24). Lepidopteran richness showed a similar monthly pattern for the two tree species evaluated (F_(3,48)_ = 1.79, p> 0.16). Interaction between habitat and month was significant (F_(6, 48)_ = 6.15, p<< 0.001), revealing that the non-excluded plots had a significantly lower lepidopteran richness in July and September whereas, in August and October, richness was similar among the three habitats (Fig 2). The interaction between species and habitat was not significant (F_(6,48)_ = 0.57, p>0.76).

### Lepidopteran abundance

**Fig 3:**
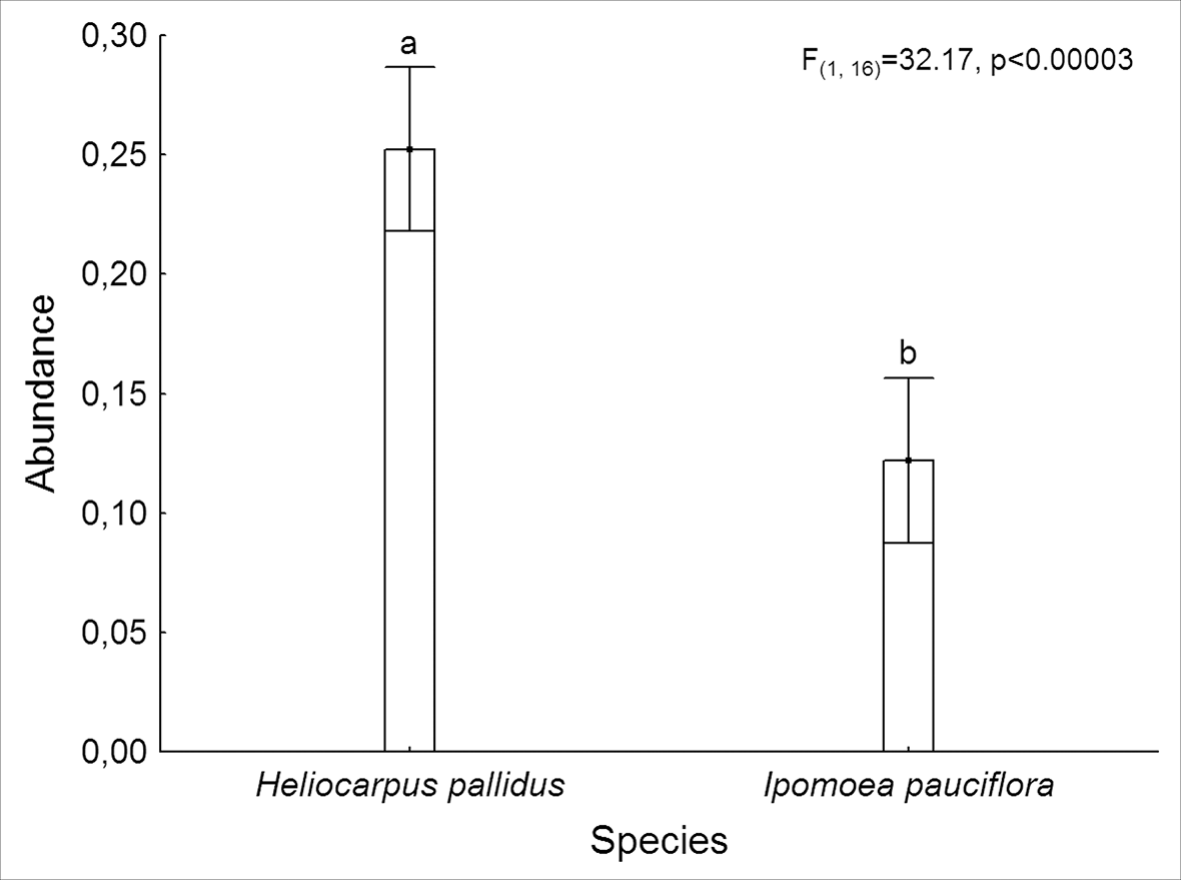
Lepidopteran abundance associated with tree species. Abundance (Log10 (number of individuals per tree + 1) of lepidopteran larvae associated with the trees *H*. *pallidus* and *I*. *pauciflora* in a seasonal forest in Sierra de Huautla, Morelos, Mexico. Vertical lines represent a 95% confidence interval.

**Fig 4:**
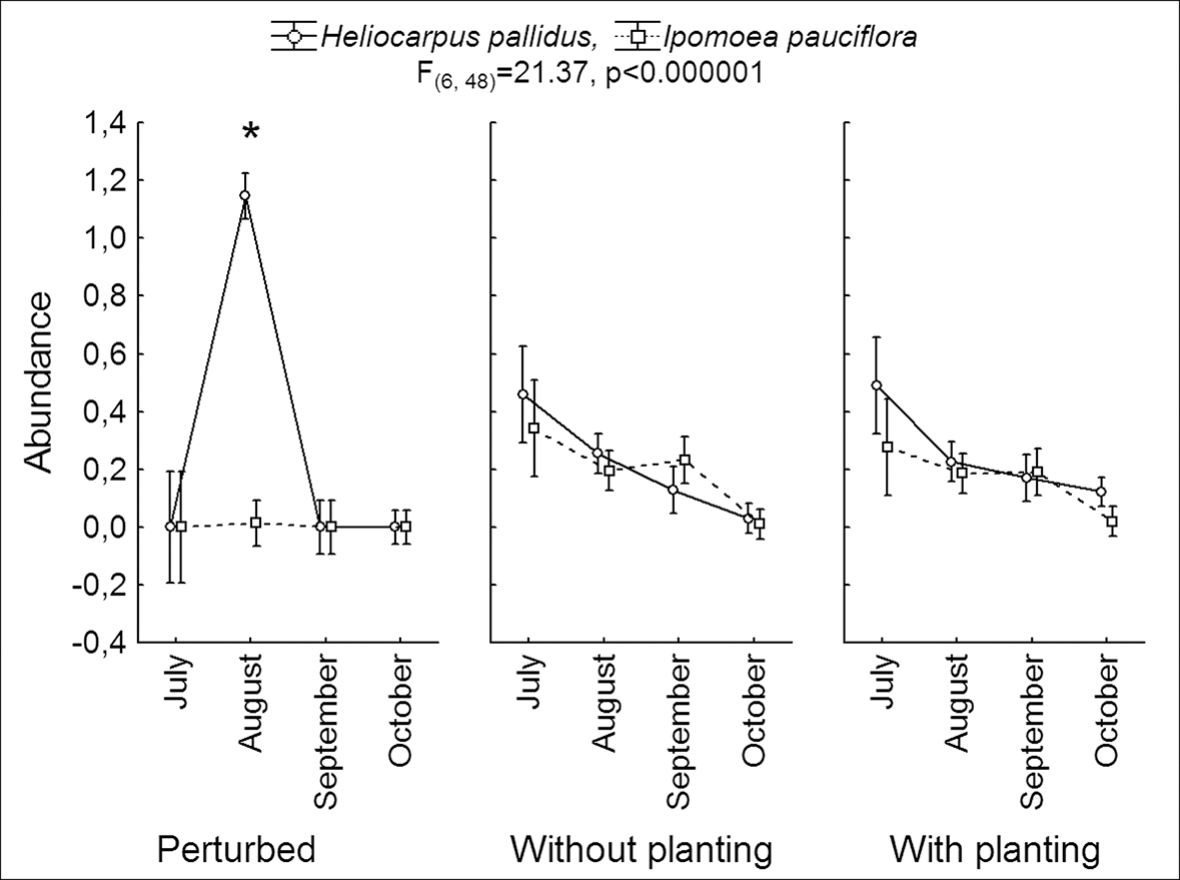
Lepidopteran abundance associated with tree species in different experimental treatments. Abundance (Log10 (number of individuals per tree + 1) of lepidopteran larvae associated with trees of *I*. *pauciflora* and *H*. *pallidus* in excluded sites, with and without plantations, and perturbed sites with access for livestock during the rainy season from July to October 2010 in the seasonal forest of Sierra de Huautla, Morelos, Mexico. Vertical lines represent 95% of the data. The asterisk represents significant differences evaluated with post hoc Tukey test, mainly due to the super abundance of *A*. *armida*.

Lepidopterans were almost four times more abundant in *H*. *pallidus* (15.61 ± 4.98 individuals) compared to *I*. *pauciflora* (4.32 ± 0.76 individuals; F_(1, 16)_ = 32.17, p<< 0.001; Fig 3). Lepidopteran abundance was similar in the three habitats (F_(2, 16)_ = 3.08, p = 0.07). Statistical analysis showed that lepidopteran abundance differed over the course of the four months (F_(3,48)_ = 47.96, p<<0.001). The post hoc Tukey test revealed that the plants had a higher and similar abundance during July and August (12.41 ± 2.78 and 22.59 ± 8.95 individuals respectively), while abundance was intermediate in September (3.95 ± 0.76 individuals) and lowest in October (0.91 ± 0.37 individuals). Interaction between species and habitat was significant (F_(2, 16)_ = 10.70, p<0.001) and the post hoc Tukey test revealed that *H*. *pallidus* presented a similar lepidopteran abundance in all three habitats, whereas *I*. *pauciflora* presented a lower lepidopteran abundance in the non-excluded plots (Fig 4).

### Similarity index

**Fig 5:**
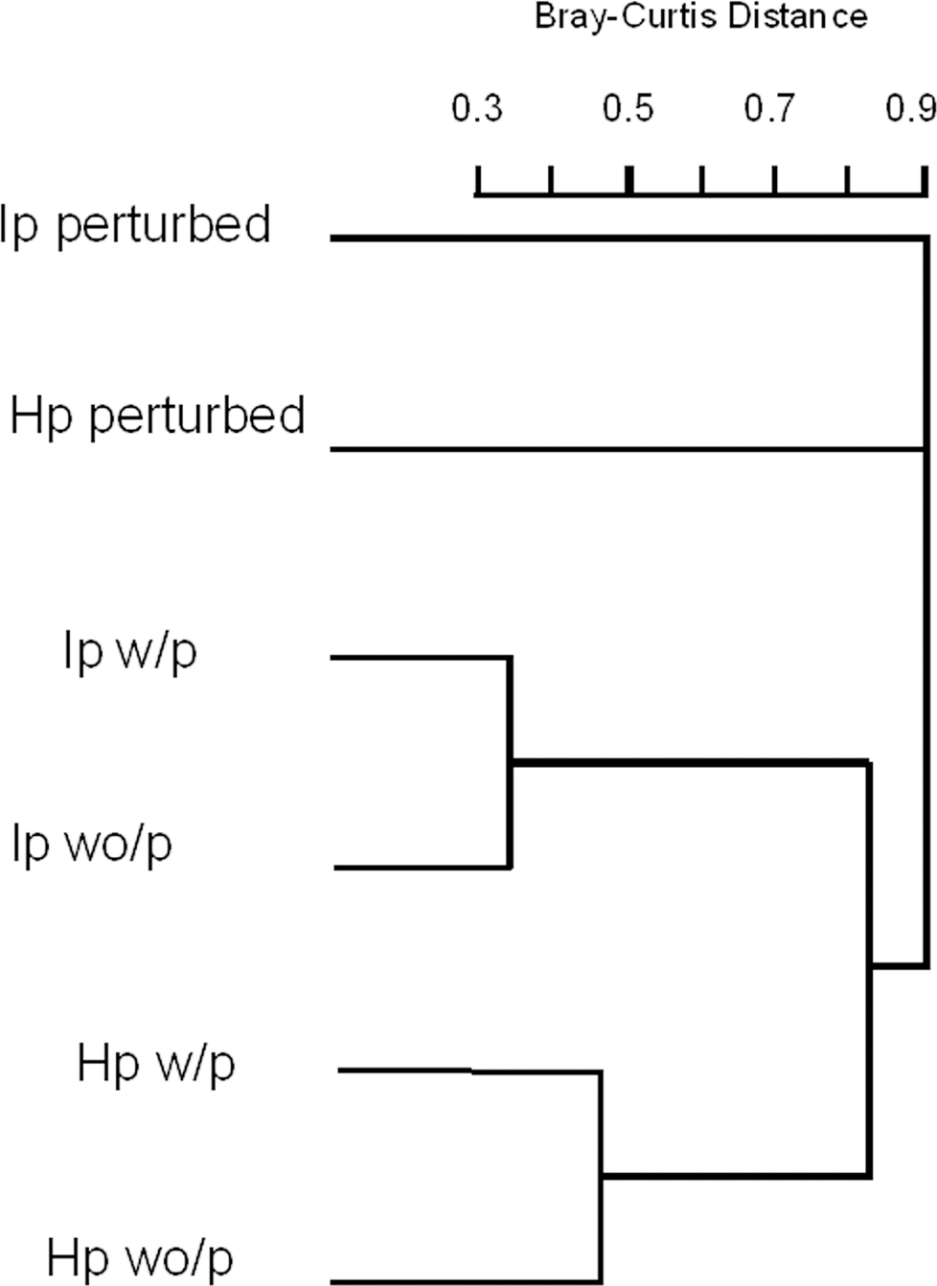
Bray- Curtis similarities between Lepidopterans associated with different plant species and experimental treatments. Dendrogram showing Bray-Curtis similarities in lepidopteran community between host plants and sites. Ip = *I*. *pauciflora*, Hp = *H*. *pallidus*; perturbed = control sites with cattle grazing; w/p = excluded plots with plantation; wo/p = excluded plots without plantation

Similarity analysis of the lepidopteran community showed a tree with three main branches; the first and the second grouped Lepidopteran communities associated with *H*. *pallidus* and *I*. *pauciflora* growing in perturbed plots, the third branch grouped Lepidopterans associated with plants in cattle-excluded plots and it is further subdivided in the communities associated with *I*. *pauciflora* or *H*. *pallidus*. A Mantel permutation test (100 permutations) showed the dendrogram to be significant (r = 0.98, p = 0.02, Fig 5).

### Herbivory

**Fig 6:**
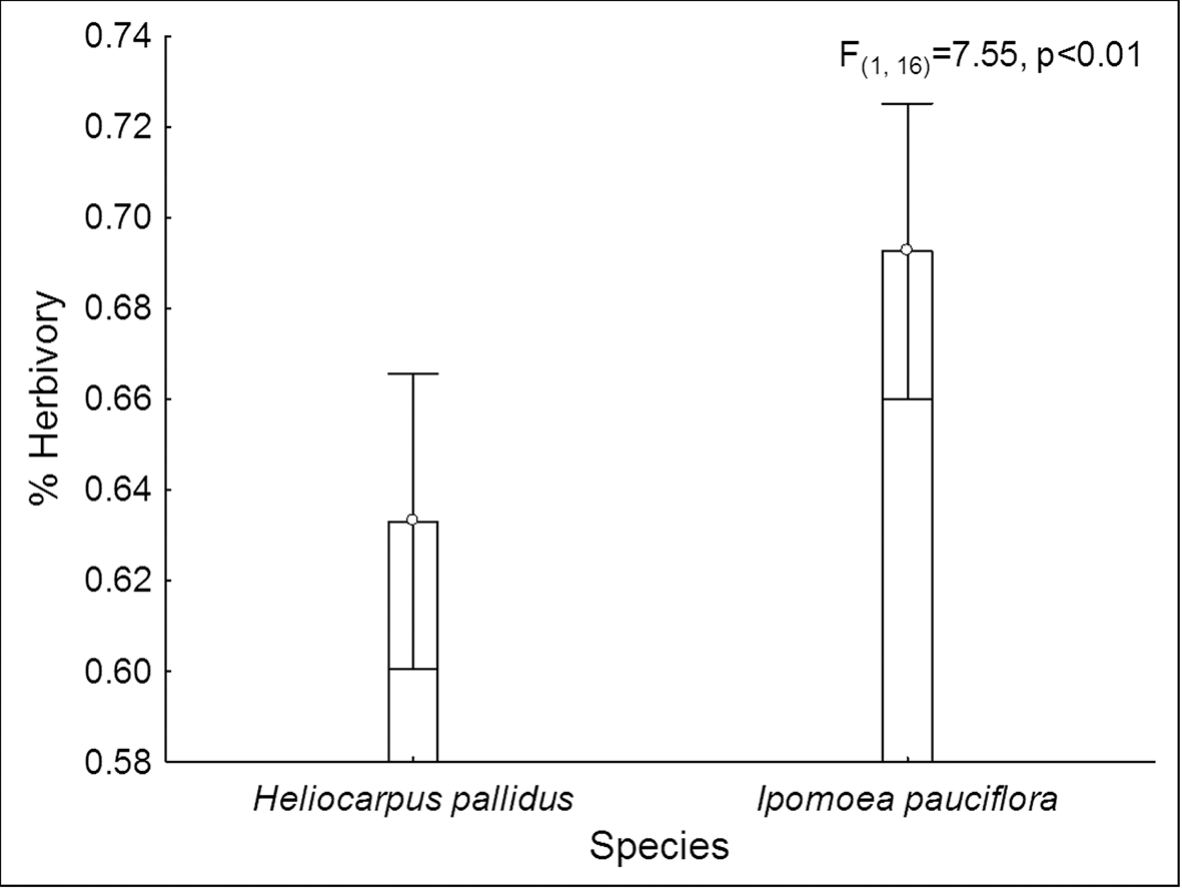
Herbivory in *I*. *pauciflora* and *H*. *pallidus*. Herbivory (arcsin square root transformed (proportion of leaf area removed +0.1)) recorded in plants of *I*. *pauciflora* and *H*. *pallidus* during the rainy season of 2010 in the tropical dry forest of Huautla, Morelos, Mexico. Vertical lines represent a 95% confidence interval.

The percentage of accumulated herbivory was significantly higher in *I*. *pauciflora* (30.81 ± 1.38%) than in *H*. *pallidus* (26.14 ± 0.96%; F_(1,16)_ = 7.55, P < 0.01; Fig 6). Herbivory did not differ significantly among habitats (F_(2,16)_ = 1.14, P > 0.34) and the interaction between species and habitat was also non-significant (F_(1,16)_ = 2.22, P > 0.14).

## Discussion

Ecosystem restoration is urgently required in many places of the world where land degradation is pervasive. The tropical dry forests (TDF) of Mexico have suffered greatly from deforestation and land use change over the last 50 years and at present only 37% of the original area remains in good condition [40,45]. Since TDFs in Mexico contain 20% of the national biodiversity [46], the recovery of such ecosystems is a national priority; however, restoration in TDFs in Mexico is still incipient and few examples are cited in the literature [5,35,47,48].

In this study, we evaluated a restoration project experience that began in 2006, where cattle were excluded from several plots and a tree enrichment treatment was incorporated. We found that cattle exclusion is crucial to the return of lepidopteran diversity to the area; the fenced plots had 20 times more lepidopteran species than the control cattle grazed sites. Other studies evaluating forest restoration have also found that lepidopterans are able to recover following restoration; Hernández et al. [5] found that after eight years of restoration in a TDF on the Mexican Pacific coast, the lepidopteran community was similar to that of mature forests. In other ecosystems, Waltz and Covington [10] restored the understory of a ponderosa-pine forest and found a 3.5% increase in butterfly species richness after 3 years of treatment. Several studies have evaluated whether selectively logged tropical forests could host butterfly communities similar to those of pristine forests and have found that, although species richness per family was equivalent, species composition replacement took place [49,50]. In this study, we also found species replacement between sites (high beta diversity). Other studies assessing the impact of human intervention in forests using butterflies as indicators have found that members of the family Nymphalidae (Satyrinae and Morphinae) in Borneo were absent in logged forests [51], highlighting the importance of habitat heterogeneity for community conservation. Summerville et al [8,9] compared lepidopteran community assemblages in restored prairies of different ages and found very high inter-year variation but that the rarefaction curves showed that older restored site diversity converges with that of undisturbed sites. However, in a previous study, Holl [7] argued that the utility of adult butterflies as indicators of restoration may be limited because of their high mobility. Lepidopteran differences in restored *vs*. perturbed sites when plant food is available may be related to predator abundance [52,53] and/or changes in microenvironmental conditions, particularly temperature increases and humidity decreases with perturbation [23,54,55]. The higher richness in restored sites of lepidopteran larvae when their mobility is minimal represents an increase in the habitat quality and availability of resources for this group of insects.

In this study, fencing against cattle was more important than enrichment plantings for the lepidopteran community; there were no significant differences between lepidopteran richness and abundance under natural successional *vs*. manipulated succession with plantings. Surprisingly, the most abundant species was not found within the fenced area where higher lepidopteran diversity was found, but in the control area where cattle were grazing. The species *A*. *armida* was favored by disturbance in *H*. *pallidus*. Coincidently this species is reported to be used as food by local people [56]; larvae are harvested during the wet season and consumed. *Arsenura armida* is highly appreciated because its larvae are large (10cm) with high protein content (51.3%) and occur in large numbers [56]. In the state of Veracruz, Mexico, *H*. *pallidus* trees are left to grow in perturbed areas to ensure a harvest of *A*. *armida* [57]. In this case, *A*. *armida* could be considered an indicator of disturbance, and its higher abundances may impede other lepidopterans from establishing in colonizing trees.

Lepidopteran community composition associated with the two tree species studied confirmed that lepidopterans select their food according to host plant identity more than habitat type. The similarity analysis showed that lepidopteran communities associated with *I*. *pauciflora* are grouped and separated from those associated with *H*. *pallidus*. Another study with lepidopteran larvae comparing natural succession *vs*. restored sites found similar results in the tropical dry forest of Chamela [5]; moths and butterflies select food for their young according to plant identity regardless of habitat quality. Chemical plant composition appears to dictate plant-herbivore associations independently of site conditions [17], therefore if lepidopteran population sources exist in the surrounding (conserved) areas, herbivores can arrive to the restored site following establishment of their host plants. Since we only studied lepidopterans associated with two tree species, more investigations considering larvae associated with all tree species are required to determine the response of the whole lepidopteran community.

Temporal patterns of lepidopteran richness and abundance followed the rainy season; a greater number of species and individuals were found at the beginning of the season and then presented a constant decline throughout the season, with the exception of plants in the perturbed sites where associated lepidopteran richness was constant since only one species was found. Lepidopteran diversity explored in other tropical dry forests of Mexico has been found to present a peak in abundance and richness in the middle of the rainy season [58], thus contrasting with the findings of this study where the peak occurred at the beginning of the rainy season; this may be due to the difference in precipitation since in Huautla there is 100 mm less rain than in Chamela.

### Herbivory

Apart from Lepidopteran diversity, this study showed that herbivory rates were different between plant species. *Ipomoea pauciflora* had higher levels of herbivory regardless of its reportedly strong chemical defenses [59]. A specialist herbivore may therefore be responsible for the observed removal of plant tissue [60]. Other studies have found that herbivory rates depend on habitat resource availability [61] because plants in richer habitats invest their resources in growth instead of defense. A study with *H*. *pallidus* in the TDF of Chamela showed higher levels of herbivory in riparian compared to deciduous habitats [62], which contrasts with our findings; however, the highest percentage of leaf removal in Chamela (26%) was similar to our results (25%). In this study, both species showed similar herbivory rates throughout the sites, implying that a) environmental resources are similar between studied habitats and plants have similar nutritional characteristics, or b) the lepidopteran communities differ among sites. The mechanisms behind this pattern remain to be explored. It is interesting that we found a peak in lepidopteran abundance in August in the *H*. *pallidus* plants growing in perturbed sites, but this peak did not translate into an increased herbivory rate in those plants. It is possible that the plants compensated for herbivore attack by producing more leaves throughout the season [63] and, since we only measured leaf consumption at the end of the year, we may have missed these more subtle differences in the patterns of herbivory.

Regarding restoration, this study shows that lepidopteran larvae attracted to sites with enriched plantings perform similarly to those attracted to sites with natural recruitment and planting is therefore an appropriate strategy for the more rapid incorporation of biodiversity into perturbed areas. While lepidopterans in restored sites showed no difference with successional plots, investigations with other groups have found that more species are arriving to the restoration plots [32]. Vertebrates are visiting restored sites more frequently and dispersing more seeds from different species [64], therefore increases in plant diversity will eventually translate in higher lepidopteran diversity. The entire lepidopteran community associated with all plant species under the different management treatments remains to be explored in order to corroborate our hypothesis. This information would provide tools with which to accelerate ecosystem restoration including other trophic guilds.

Studies of the impact of cattle ranching activities in tropical dry forest suggest the exclusion of cattle in order to favor ecosystem recovery [2,48,65,66], and therefore cattle grazing should be prevented. In our study, following four years of cattle exclusion and the establishment of plantings, lepidopteran richness was found to have increased 20-fold in the excluded plots compared to perturbed areas, whereas herbivory levels were equally high in restored and perturbed sites. Restoration was a successful strategy for attracting lepidopterans and cattle exclusion was the main factor that explained lepidopteran diversity

## Supporting Information

S1 Table Abundance of each identified lepidopteran morphospecies associated with *H*. *pallidus* and *I*. *pauciflora* in Sierra de Huautla, Morelos, Mexico. (DOCX)
