# Supplementary material for: Recovering More than Tree Cover: Herbivores and Herbivory in a Restored Tropical Dry Forest
Source: PLoS One. 2015 Jun 1;10(6):e0128583. doi: 10.1371/journal.pone.0128583 (PMC4452621; doi:10.1371/journal.pone.0128583)
Supplement: S1 Table — (DOCX) [file pone.0128583.s001.docx]

| **Family** | **Lepidopteran species** | **Abundance** | ***Heliocarpus pallidus*** | ***Ipomoea pauciflora*** |
| --- | --- | --- | --- | --- |
| **Apatelodidae** | *Apatelodes sp.* | 1 | ✓ |  |
|  | *Apatelodes sp.* | 1 | ✓ |  |
| **Arctiidae** | *Hypercompe suffusa* | 32 | ✓ | ✓ |
|  | *Euchaetes egle* | 1 | ✓ |  |
| **Crambidae** | *Phostria tedea* | 61 |  | ✓ |
|  | *Conchylodes sp.* | 50 | ✓ | ✓ |
| **Geometridae** | Geometridae sp1 | 9 | ✓ |  |
|  | Geometridae sp2 | 25 | ✓ |  |
|  | Geometridae sp3 | 10 | ✓ |  |
|  | Geometridae sp4 | 2 | ✓ |  |
|  | Geometridae sp5 | 3 | ✓ |  |
|  | Geometridae sp6 | 1 | ✓ |  |
|  | Geometridae sp7 | 1 |  | ✓ |
|  | Geometridae sp8 | 1 | ✓ |  |
|  | Geometridae sp9 | 1 |  | ✓ |
| **Hesperiidae** | Hesperidae sp1 | 4 | ✓ |  |
|  | Hesperidae sp2 | 1 | ✓ |  |
|  | Hesperidae sp3 | 1 |  | ✓ |
| **Lymantridae** | *Orgyia* sp. | 10 | ✓ | ✓ |
| **Noctuidae** | Noctuidae sp1 | 30 | ✓ |  |
|  | *Anomis sabulifera* |  | ✓ |  |
|  | Noctuidae sp2 | 1 | ✓ |  |
|  | *Protogygia album* |  | ✓ |  |
| **Psaphididae** | *Psaphida rolandi* | 3 |  | ✓ |
| **Psychidae** | Psychidae sp1 | 1 |  |  |
| **Saturniidae** | *Automeris io* | 1 | ✓ |  |
|  | *Arsenura armida* | 422 | ✓ |  |
|  | *Citheronia sp.* | 4 |  | ✓ |
| **Sphingidae** | *Manduca rustica* | 1 | ✓ |  |
